# Interactions Between Different Age-Related Factors Affecting Balance Control in Walking

**DOI:** 10.3389/fspor.2020.00094

**Published:** 2020-07-31

**Authors:** Hendrik Reimann, Rachid Ramadan, Tyler Fettrow, Jocelyn F. Hafer, Hartmut Geyer, John J. Jeka

**Affiliations:** ^1^Department of Kinesiology and Applied Physiology, University of Delaware, Newark, DE, United States; ^2^Institute for Neural Computation, Ruhr University, Bochum, Germany; ^3^Department of Applied Physiology and Kinesiology, University of Florida, Gainesville, FL, United States; ^4^Robotics Institute, Carnegie Mellon University, Pittsburgh, PA, United States

**Keywords:** aging, balance, modeling, neuromechanic, vision, muscle strength, cognition, walking

## Abstract

Maintaining balance during walking is a continuous sensorimotor control problem. Throughout the movement, the central nervous system has to collect sensory data about the current state of the body in space, use this information to detect possible threats to balance and adapt the movement pattern to ensure stability. Failure of this sensorimotor loop can lead to dire consequences in the form of falls, injury and death. Such failures tend to become more prevalent as people get older. While research has established a number of factors associated with higher risk of falls, we know relatively little about age-related changes of the underlying sensorimotor control loop and how such changes are related to empirically established risk factors. This paper approaches the problem of age-related fall risk from a neural control perspective. We begin by summarizing recent empirical findings about the neural control laws mapping sensory input to motor output for balance control during walking. These findings were established in young, neurotypical study populations and establish a baseline of sensorimotor control of balance. We then review correlates for deteriorating balance control in older adults, of muscle weakness, slow walking, cognitive decline, and increased visual dependency. While empirical associations between these factors and fall risk have been established reasonably well, we know relatively little about the underlying causal relationships. Establishing such causal relationships is hard, because the different factors all co-vary with age and are difficult to isolate empirically. One option to analyze the role of an individual factor for balance control is to use computational models of walking comprising all levels of the sensorimotor control loop. We introduce one such model that generates walking movement patterns from a short list of spinal reflex modules with limited supraspinal modulation for balance. We show how this model can be used to simulate empirical studies, and how comparison between the model and empirical results can indicate gaps in our current understanding of balance control. We also show how different aspects of aging can be added to this model to study their effect on balance control in isolation.

## 1. Introduction

Walking on two legs is inherently unstable and requires continuous control to keep the body upright. Learning to do so is a major developmental milestone for infants. At the opposite end of the age spectrum, it is well-known that older adults are at increased risk of falling, with a high probability of falls resulting in injury (Herdman, 1997; Kannus et al., 1999). In the US alone, 3.2 million falls occur each year leading to medical treatment, with health care costs exceeding $30 billion. The risk of falling increases with age (Rubenstein, 2006), and the injuries resulting from falls limit mobility and impair the ability to perform daily tasks, leading to a decline in quality of life (Fuller, 2000; Stevens et al., 2006).

We know that the tendency to fall more often during walking is associated with a number of factors (Osoba et al., 2019), such as weaker muscles (Pijnappels et al., 2008a), slow gait (Jerome et al., 2015), and cognitive decline (Lamoth et al., 2011), and all of these factors also tend to change with age. But we do not understand the causal relationship between these factors, and a decline in upright balance control. One major hurdle is that all of these changes happen simultaneously over a long time. As we get older, our muscles tend to slowly get weaker, we walk slower, and lose mental acuity. But what is the causal relationship, if any, between these factors? Do our weakening muscles cause us to walk slower, which is harder to control and causes increases in fall risk? Or is the slower walking a coping mechanism to account for longer cognitive processing time for executive function?

The sensorimotor control loop for walking integrates dynamic processes ranging over sensation, neural processing, integration with an overall motor plan, transformation into descending motor commands, reflex arcs in the spinal cord, muscle physiology, and force generation and biomechanical interaction with the environment. The processes at all of these levels are dynamically coupled and can potentially interact and affect each other. Furthermore, slow changes in one factor could drive adaptive changes in other processes, like preferring a slower walking speed, to offset the increased risk of falls from longer reaction times, or to account for increased fatigability (Finsterer and Mahjoub, 2014). This integration on a developmental time scale makes it hard to isolate the mechanisms of how each single factor affects balance control and fall risk.

Here we argue that the appropriate tool to solve this problem of isolating and understanding the effect of individual fall risk factors on balance control is to develop a computational model that encompasses the dynamics of the neuromechanical processes at each level. In the following we will frame balance control as a sensorimotor control problem that is solved by the central nervous system, and summarize recently published experimental results from studies with sensory perturbations that measure how this neural control system works (section 2). We will then discuss correlates for deteriorating balance control in older adults, with a focus on muscle weakness, slow walking, cognitive decline, and increased dependency on visual information and review evidence for how they affect fall risk from the general literature, combined with a review of recent empirical findings on how some of these factors affect neural feedback control mechanisms (section 3). We will make the case that the appropriate method to understand the interaction between these different factors and balance control is to use predictive, computational models of balance control, and introduce an existing neuromechanical model of human walking that can be physiologically “aged” to serve as a basis for understanding age effects. We close by performing a simulation study to demonstrate how such predictive models can be used to understand interactions between age-related factors and balance control and to test the functional validity of hypotheses that are hard to evaluate experimentally (section 4).

## 2. Sensorimotor Control of Balance in Walking

A walking human is a mass moving through space, accelerated by forces from muscles, gravity and interaction with the ground. The general walking pattern of moving forward and setting one leg in front of the other emerges passively to some degree, from the mechanical structure of the body. This has been demonstrated by passive walkers, legged mechanical devices that spontaneously generate stable walking patterns, requiring only mechanical energy to off-set losses from friction, usually from walking down an inclined plane (Collins, 2005). While the human body shares some general characteristics with passive walkers, mechanical analysis has shown that it is not mechanically stable. A passively walking human body will generally fall over sideways after a few steps (Kuo, 1999). Stable walking requires a regulating process that actively maintains upright balance. Here we focus on the frontal plane, where balance control is more demanding than in the sagittal plane (Bauby and Kuo, 2000).

Active control of upright balance during walking requires a sensorimotor control loop that collects sensory information about the movement of the body through space, detects deviations from the upright posture, and generates appropriate muscle forces to correct these deviations. To detect deviations from the upright posture, the nervous system uses mainly the proprioceptive, vestibular and visual systems (Shumway-Cook and Horak, 1986). Research in standing balance control has shown that information from these different sensory modes is combined, or “fused,” into an estimate of the mechanical state of the whole body in space. If this estimate detects a deviation from the upright, the control loop sends descending motor commands that change muscle activation to generate a corrective force. In quiet standing, this force is usually generated mainly by the ankle musculature that pull on the body as a single, rigid rod, but in situations with substantial sway, the hip joint gets involved as well (Horak and Nashner, 1986). The body behaves, essentially, as an inverted pendulum with one or two links that is fixed to the ground and rotates around the ankle and hip joints.

The biomechanical effect of a muscle activation during walking is highly dependent on the point in the gait cycle (Reimann et al., 2019). The walking body is mechanically complex, with arms and legs moving largely independent from each other, though highly coordinated (Punt et al., 2015; Thompson et al., 2017). The general function of the sensorimotor loop for balance control is the same as in standing (Peterka, 2002), but generating force to correct a detected deviation from the upright posture is less straightforward in walking. In standing, the *gastrocnemius* muscle will always pull the body backward, but in walking, its effect changes drastically based on the gait cycle. During late double stance when the leg is trailing, the *gastrocnemius* will increase the push-off force and move the body forward (Klemetti et al., 2014; Hsiao et al., 2015). During early double stance, when the leg is leading, the *gastrocnemius* will extend the ankle and knee, pushing backward against the body (Hof and Duysens, 2018). During swing, the *gastrocnemius* only moves the foot in the air and does nothing for the whole body. Since the result of a *gastrocnemius* activation depends so strongly on the point in the gait cycle, the appropriate motor response to a detected deviation from the upright posture has to be equally phase-dependent.

**Figure 1:**
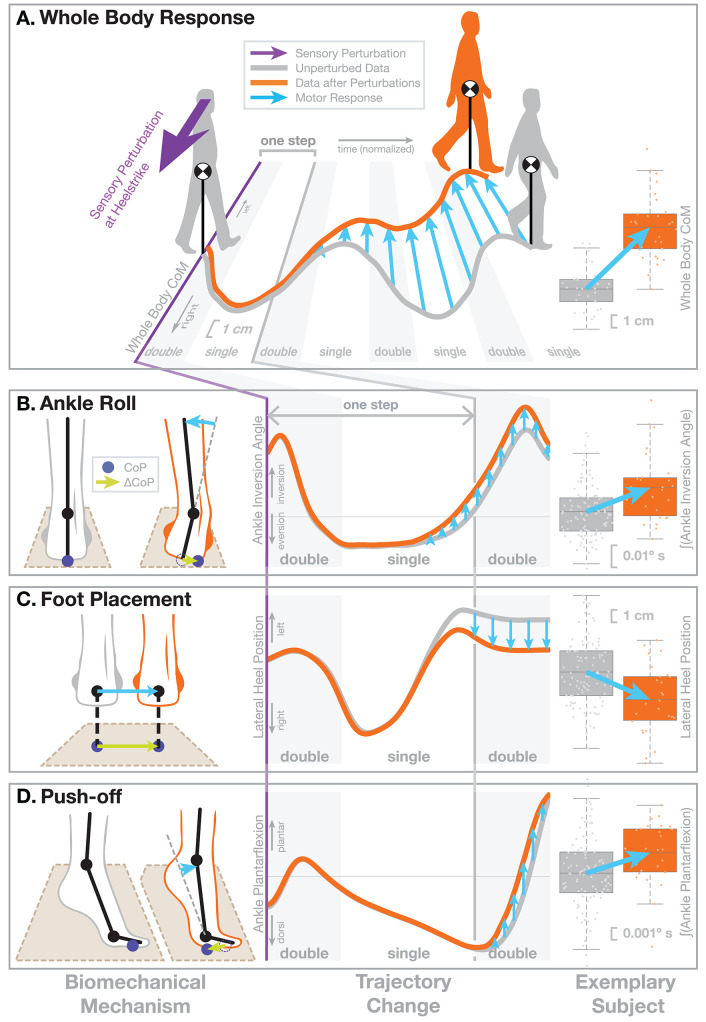
Illustration of the motor response to sensory fall stimuli. In this figure we demonstrate the biomechanical action of the different balance mechanism. We selected a non-representative sub-set of previously published data (Reimann et al., 2018b) to clearly illustrate the biomechanics. The subject receives a visual stimulus that induces the sensation of falling to the *right* (purple arrow). **(A)** This results in an overall shift of the whole-body CoM toward the *left*, i.e., the direction opposite to the perceived fall, over the next four steps. The central panel shows the average horizontal CoM trajectory of an exemplary subject for normal, unperturbed steps (gray line). In response to the visual stimulus representing a fall to the right (purple), the average CoM shifts toward the left over the following four steps (orange line). The horizontal axis represents normalized time, with gray areas indicating double stance, white areas single-stance period. Blue arrows show the difference between the two trajectories representing the motor response to the stimulus. The panel on the right shows the total CoM shift after four steps for the same subject, where boxes show the means and 25th–75th quartiles, and whiskers show the data range and dots show the individual data points. The lower part shows the three balance mechanisms that modify the ground reaction force to generate this change in whole-body CoM movement, **(B)** Ankle Roll, **(C)** Foot Placement, and **(D)** Push-Off. In each row, the **left column** shows the biomechanical changes represented by these mechanisms, illustrating how each mechanism affects the forces against the ground. The dark blue dots indicate the CoP position and the green arrows show how the CoP changes as a result of the motor response. Each mechanism results in a CoP shift to the *right*, and this force difference leads to an acceleration of the whole-body CoM to the *left* that results in the CoM trajectory shown in part **(A)**. The **middle column** illustrates changes in how a relevant kinematic variable for each mechanism. The graphs start on the heel-strike triggering the perturbation and the horizontal axis shows normalized time, ranging over the first two double stance periods (gray) and first single stance period (white), which is the period of initial response to the artificial stimulus. The gray line show averages over the normal, unperturbed trajectories for the same subject. The orange lines represent the kinematic changes observed for each mechanism, but since these changes are generally small, we manually modified the curves here to exaggerate them, for the purpose of clearly demonstrating the function. The **right column** shows the actual, unmodified data for the same exemplary subject, where the response is represented by a single aggregate variable. For Ankle Roll, the aggregate variable is the integral of the stance leg ankle inversion angle over the first post-stimulus single stance period (white in the left panel). For the Foot Placement, the aggregate variable is the lateral heel position of the leading leg at the first post-stimulus heel-strike, relative to the trailing leg heel position. For the Push-off, the aggregate variable is the integral of the trailing leg ankle plantarflexion angle over the second post-stimulus double stance period (gray in the left panel). The blue arrows in all panels indicate how each variable changes in response to the stimulus.

What motor responses do humans use when they detect a deviation from the upright, depending on the point in the gait cycle? To answer this question experimentally, we have previously developed a paradigm that perturbs a sensory system to induce artificial fall sensations in walking humans and observes the motor response using kinematics, kinetics, and electromyography (EMG). In this paper, we will summarize results from studies that used this paradigm to investigate the effect of different age-related factors on balance control. All data discussed here has been published elsewhere before. Our platform consists of an instrumented treadmill (Bertec Inc, Columbus, OH, USA) surrounded by a virtual reality (VR) environment projected onto a domed screen (see Box 1 for details). Artificial fall stimuli are triggered on heel-strike and induce the sensation of falling sideways, rotating in the frontal plane around the stance foot ankle joint during single stance. This rotation around the ankle implies a lateral translation of the whole-body center of mass (CoM). Figure 1 illustrates the motor responses to these artificial fall stimuli. The overall response is that people move their body in the opposite direction of the perceived fall (Figure 1A). After a sensory stimulus induces the sensation of a fall to the right (purple arrow), participants move their CoM (orange line) to the left over the course of the following steps, compared to how the CoM usually moves without a sensory stimulus (gray line). This is the expected response to a fall stimulus. The neural control system detects a deviation from the upright in the form of the artificial fall stimulus to the right and reacts by moving the body back to what it estimates to be upright, i.e., leftward. Since the detected deviation was not real, but artificial, the result is a leftward shift of the whole body CoM in space. By artificially inducing the sensation of a lateral fall in a controlled, repeatable manner, we can observe how the neural controller generates this whole-body leftward movement. The lower panels of Figure 1 show three different biomechanical mechanisms to modulate the lateral ground reaction force and generate a lateral force against the ground to the right that accelerates the body to the left. In the following paragraphs we will explain each mechanism according to the example of a fall stimulus to the right triggered by a right heel-strike, as illustrated in Figure 1, but note that all three mechanisms are used regardless of direction and triggering foot.

Box 1 **Virtual walking environment and artificial fall stimuli.**

**Figure:**
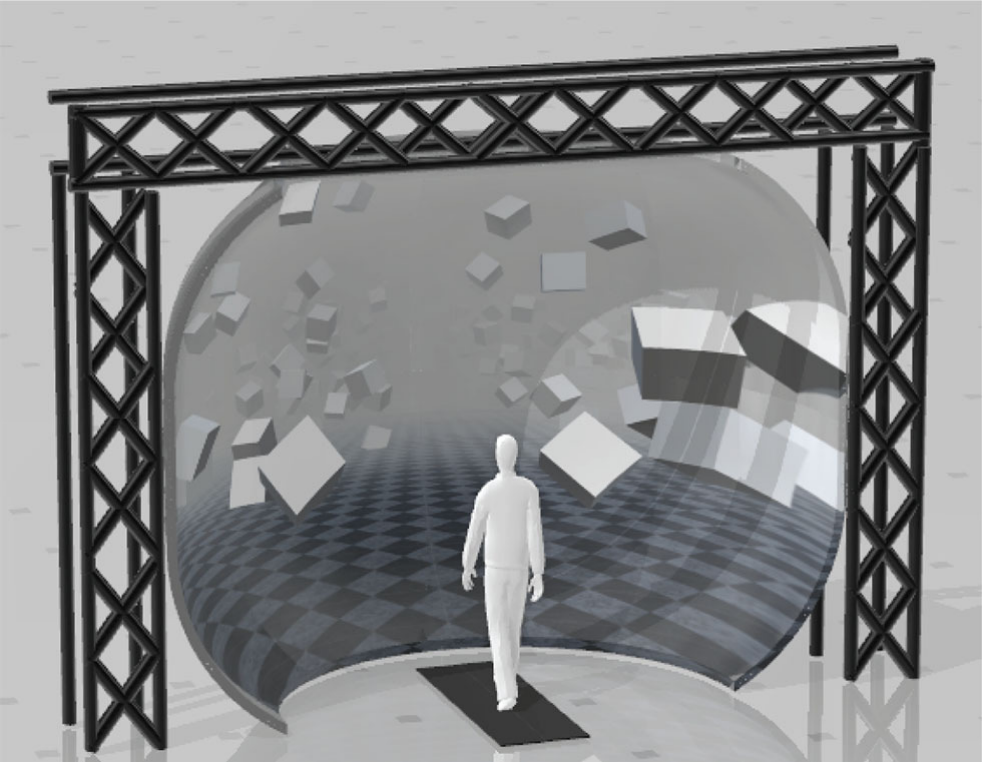

**Virtual Walking Environment**. Participants walk on a treadmill in a virtual reality (VR) environment projected onto a domed screen. The screen covers almost the complete field of vision of the participant walking on the treadmill. The participant's point of view in the virtual environment is linked to the head position in real time, measured by the motion capture system, creating a motion parallax effect. The speed of the treadmill is linked to the participant's pelvis position, using a non-linear PD-controller to keep the subject centered on the treadmill along the sagittal axis. This user-driven mode allows the participants to walk at a self-selected speed and spontaneously speed up or slow down at any time. The forward progression in the VR environment is also linked to the treadmill speed in real time. In combination, these components create an immersive VR experience, where participants walk through a virtual environment without the secondary task of matching their speed to the treadmill, and where the visual information available to the participants is determined almost exclusively by the virtual environment.

**Artificial Fall Stimuli**. To induce the sensation of a fall to the side, we stimulate either the visual or the vestibular system of the participants walking in the virtual environment. A **visual** fall stimulus consists of the virtual world rotating around the sagittal axis through the center of the treadmill. This rotation generates optical flow on the participant's retina that is similar to the optical flow of falling sideways by rotating around the stance foot ankle joint. The velocity of this rotation increases at a constant rate of 45–90° s^−2^, depending on experimental paradigm, for 600 ms, resulting in a horizon tilt of ≈7–15°. The scene remains fixed at that tilt for 2 s, then resets at a constant rate over 1 s to prepare for the next stimulus. A **vestibular** fall stimulus is induced using Galvanic vestibular stimulation (Fitzpatrick and Day, 2004). A light electric current is delivered between two electrodes attached to the mastoid processes behind the ears. The current affects the vestibular nerve and induces the feeling of swaying to the side, with the direction depending on the polarity of the signal. We use a square wave signal with 0.5–1 mA amplitude and 600–1,000 ms duration, depending on the experimental paradigm. Fall stimuli are generally triggered on heel-strike and are followed by a wash-out period of variable length.

### 2.1. Ankle Roll

Ankle roll is an active ankle inversion torque at the stance leg in single stance (Figure 1B). This torque inverts the ankle by pulling the foot segment and the rest of the body together. The foot segment rolls on the ground, shifting the CoP to the right. The upper body is accelerated in space to the left. This mechanism is analogous to the ankle strategy in standing balance control (Horak and Nashner, 1986). This mechanism was first noted in walking by Hof et al. (2007) and later confirmed as an active mechanism (Hof et al., 2010; Reimann et al., 2017, 2018b; Hof and Duysens, 2018). The roll torque is generated by an activation increase in the medial ankle muscles (*tibialis anterior, gastrocnemius medialis*) and a decrease in the lateral muscles (*peroneus longus*) (Reimann et al., 2018b; Fettrow et al., 2019). We quantify this mechanism by integrating the difference in the subtalar joint angle between the perturbed and unperturbed steps over single stance.

### 2.2. Foot Placement

Foot placement is an active shift of the lateral foot placement location at heel-strike (Figure 1C). This shift in foot position changes the lever arm of the gravitational force acting on the body through the new stance leg during the following step. When detecting a fall to the right, the foot placement is shifted to the right, so gravity pulls the body mass more to the left. This mechanism was first introduced in robotics by Townsend (1985), who showed that foot placement modulation is already sufficient to control upright balance in a walking humanoid. It was discussed later both in robotics (Kuo, 1999; Pratt et al., 2006) and human motor control (Bauby and Kuo, 2000; Hof, 2008) and is now widely accepted to be one of the dominant mechanisms for human balance control during walking (Wang and Srinivasan, 2014; Bruijn and van Dieën, 2018; Reimann et al., 2018a). The lateral shift of the left foot before heel-strike can be generated by a left hip abduction, but also by a combination of internal rotation of the stance leg knee and external rotation of the swing leg hip joint, and we have found evidence for both (Reimann et al., 2018b; Fettrow et al., 2019). We quantify the foot placement shift by calculating the difference between the perturbed foot placement and the predicted foot placement based on the CoM position and velocity at mid-swing using a linear model fitted to the unperturbed steps (for details see Wang and Srinivasan, 2014; Bruijn and van Dieën, 2018; Reimann et al., 2018b).

### 2.3. Push-Off Modulation

Push-off modulation is a change in the plantar-dorsiflexion angle of the trailing leg during double stance, starting in late single stance (Figure 1D). After the right heel-strike triggers a fall stimulus to the right, the right ankle plantarflexion increases, pushing more strongly in the subsequent double stance. This increased push-off shifts the body weight between the two stance legs, in a direction that is largely forward, but also to the left. Push-off is a well-known mechanism for balance in the sagittal plane, used mainly for trip recovery (Pijnappels et al., 2005, 2008b). For medial-lateral balance, this mechanism was first discussed by Kim and Collins (2013), who later showed that an ankle prosthesis using this control principle can reduce the metabolic cost of walking (Kim and Collins, 2015). We observed this mechanism in healthy young humans (Reimann et al., 2018b). The increased plantarflexion in the trailing leg is preceded by increased activity in the *gastrocnemius medialis* in late single stance (Fettrow et al., 2019). We quantify this mechanism by integrating the difference in the ankle plantarflexion angle between the perturbed and unperturbed steps over double stance.

Ankle roll, foot placement shift, and push-off modulation are three biomechanical mechanisms to change the ground reaction force and push the body to the left in response detecting a fall to the right. They become available at different times during the gait cycle and temporally coordinated by the neural control system, which shifts the response between mechanisms as they become available to generate a functional, whole-body balance response to a detected fall (Reimann et al., 2019). In section 3.5, we will review how these balance mechanisms interact with age-related factors affecting fall risk.

## 3. Effects of Aging on Balance Control

People tend to fall more often as they get older, and the probability that a fall results in injury is increased with age (Herdman, 1997; Kannus et al., 1999). In the US alone, 3.2 million falls occur each year leading to medical treatment, with health care costs exceeding $30 billion. The risk of falling increases with age (Rubenstein, 2006), and the injuries resulting from falls limit mobility and impair the ability to perform daily tasks, leading to a decline in quality of life (Fuller, 2000; Stevens et al., 2006). Many studies have identified risk factors that predict falls in older adults to some degree (Osoba et al., 2019). In this section, we review how cognitive function, muscle weakness, walking speed and increased dependency on visual information are associated with fall risk, and how these factors are related to the sensorimotor control of balance.

### 3.1. Cognitive Function

Much of the age-related cognitive decline literature has been focused on cognitive (Reuter-Lorenz et al., 2010) or general motor function (Carson, 2018). The disparity of research linking cognition and mobility may be a result of perspective of the researchers in their respective fields, with little overlap between fields. In the field of robotics, bipedal locomotion can be reproduced with passive dynamics (Kuo, 1999), calling into question the need for neural control, let alone the role of supraspinal circuits. Observation of patients in the medical field yield a different perspective, where locomotion typically deteriorates in the event of a stroke (Dean and Kautz, 2016) or Parkinson's Disease (Curtze et al., 2016) leading to the conclusion that supraspinal control is critical to the task of balance and locomotion. Postural control requires active modulation of muscle activity even in standing (Morasso and Sanguineti, 2002), and there is a wide range of evidence that cognitive processing is required.

Dual-task paradigms have been the main experimental methodology linking cognition to the control of balance and locomotion (Woollacott, 2000; Li and Lindenberger, 2002; Horak, 2006). Cognitive tasks performed during standing or walking generally interact with balance control, often leading to impaired performance in balance (Barra et al., 2006) or the cognitive task (Andersson et al., 2002). Since the 1980s, dual-task paradigms have been used to assess the role of cognition during standing (Cordo and Nashner, 1982; Stelmach et al., 1990). The results of these early studies of dual-task postural control reveal that, in general, postural stability is prioritized over the secondary task. This interaction between balance and secondary cognitive tasks is consistently stronger in older adults compared to younger (Teasdale and Simoneau, 2001; Redfern et al., 2002; Melzer and Oddsson, 2004; Lamoth et al., 2011; Schaefer et al., 2014; Li et al., 2018), as well as in populations with neuromotor impairments (Camicioli et al., 1997; Lapointe et al., 2010; Bahureksa et al., 2016). The degree to which performance in the secondary task diminishes (dual-task cost), is dependent on the perceived risk of injury (Shumway-Cook et al., 1997), indicating a complex relationship between task prioritization, perception of self, and the environment (Wrightson and Smeeton, 2017). Moreover, the specific kind of secondary task influences attentional demands, with visual or arithmetic tasks having a stronger effect than verbal or auditory tasks (Beauchet et al., 2005a,b).

Interventions targeting cognition are also insightful for understanding the role of cognition in the control of balance and locomotion. Balance and strength training while performing simultaneous cognitive tasks can improve performance in dual-task protocols (Hiyamizu et al., 2012). Performing cognitive training in isolation shows transfer to balance related outcomes (Li et al., 2010). Reduced prefrontal brain activity in a walking task after a dance intervention in healthy older adults (Eggenberger et al., 2016) and a treadmill dual-task training intervention for people with Parkinson's (Maidan et al., 2018) indicates less attentional resources are dedicated to the task after the intervention. These results can help confirm a link between cognition and control of balance and locomotion.

In general, these results suggest walking becomes less automatic as age increases, shifting from spinal level control to supraspinal control (Clark, 2015). The shift of control is typically observed as prefrontal over-activation during steady state walking in older adults and other populations with hindered mobility, such as stroke (Mihara et al., 2007), Parkinson's disease (Maidan et al., 2016), and multiple sclerosis (Hernandez et al., 2016). Tasks with a cognitive aspect during walking, such as a precision stepping, have been found to increase activity in the prefrontal cortex, as measured by functional near-infrared spectroscopy (fNIRS, Koenraadt et al., 2014). Increased prefrontal activation is also observed in older adults, compared to younger adults, when increasing the difficulty of walking by adding obstacles to the environment (Chen et al., 2017; Mirelman et al., 2017). When encountering an obstacle during walking, the normal, steady state motor plan must be inhibited, requiring the nervous system to plan a new trajectory that avoids the obstacle (Potocanac et al., 2014a). This process is time-sensitive (Potocanac et al., 2014b) and older people generally perform worse (Potocanac et al., 2015). The direct assessment of supraspinal components during the actual task of interest (walking, walking over obstacles, walking on different terrain) provides the most compelling evidence that supraspinal circuits contribute to the task of balance.

### 3.2. Muscle Weakness

One of the most consistent risk factors for falling is muscle weakness (Rubenstein, 2006; Pijnappels et al., 2008b), especially in the lower limbs (Moreland et al., 2004). Declines in lower-extremity muscle strength become apparent in the 5th or 6th decade of life (Murray et al., 1980; Lindle et al., 1997), with estimated rate of strength loss of 2–3% per year in adults over age 65 (Skelton et al., 1994). Age-related strength losses are most severe at faster muscle contraction velocities (Callahan and Kent-Braun, 2011), potentially limiting older adults' ability to reposition limbs or generate force fast enough to prevent a fall in response to an unexpected perturbation.

Muscle weakness has repeatedly been correlated with both greater incidence of falls and greater incidence of factors thought to be risk factors for falls. Adults over age 65 have been found to have weaker lower-extremity muscles and greater postural instability (Hurley et al., 1998), with greater weakness being associated with poorer stability (Hasson et al., 2014; Menant et al., 2017; Gadelha et al., 2018b). Further, weaker older adults have a higher incidence of falls than their stronger counterparts (Menant et al., 2017; Gadelha et al., 2018a; Yeung et al., 2019). However, the extent to which muscle strength directly counters fall risk is unclear.

While muscle strength begins to decline in the 40s, increased incidence of falls is generally not reported as a major health concern until after age 65 (Peel, 2002). This suggests that there is either a minimum threshold of strength needed to maintain balance or that, beyond a certain point, the parallel decline of multiple physiological systems makes avoiding falls difficult. Decreased muscle strength can certainly contribute to increased fall risk, as acute muscle fatigue induces gait changes indicative of poorer stability or greater fall risk even in young healthy adults (Barbieri et al., 2014). Acute fatigue of healthy older adults leads to changes in gait and posture toward patterns seen in fall-prone older adults (Helbostad et al., 2007; Egerton et al., 2009; Foulis et al., 2017). These increases in markers of fall risk with acute muscle weakness support some direct role of muscle strength in balance.

Interventions designed to improve muscle strength can increase scores on clinical balance tests (Hess and Woollacott, 2005), reduce fear of falling (Gusi et al., 2012) and decrease fall risk (LaStayo et al., 2003), especially as part of a multifactorial approach (Sherrington and Tiedemann, 2015). Exercise interventions as a whole reduce the rate of falls by 23%, with multifaceted interventions (i.e., balance, functional, and resistance exercise) reducing the rate falls by more than 30% (Sherrington et al., 2019). However, resistance training alone may not lead to reductions in falls (Sherrington et al., 2019). Because muscle weakness is likely one of several factors leading to increased fall risk with age, there is still limited evidence of the power of muscle weakness to predict and of strength training to prevent falls (Pizzigalli et al., 2011; Granacher et al., 2013). An understanding of the mechanisms behind changes in strength and the interaction of changes in strength with other physiological systems is needed.

### 3.3. Walking Speed

Reduced walking speed has also been tied to incidence of falls (Abellan Van Kan et al., 2009; Verghese et al., 2009; Middleton et al., 2016; Geerse et al., 2019) and fear of falling (Callisaya and Verghese, 2018; Geerse et al., 2019; van Schooten et al., 2019) in older adults. Slowed gait speed is a common characteristic of aging (Himann et al., 1988; Nigg and Skleryk, 1988; Bohannon, 1997; Jerome et al., 2015). Further, declines in walking speed are correlated to declines in muscle strength (Bassey et al., 1988; Bendall et al., 1989; Rantanen et al., 1998).

Despite correlations between walking speed and falls risk, we do not understand how walking speed influences stability, balance, or falls. Greater variability and instability are thought to be indicators of greater fall risk in older adults, and many studies have tested the effects of age, walking speed, or age and walking speed on these parameters. Older adults generally have greater variability and instability than young adults (Hausdorff et al., 2001; Kang and Dingwell, 2008a,b; Verghese et al., 2009) and, because older adults typically walk slower than young adults, it has been thought that these measures of fall risk are mechanistically related to walking speed. However, associations between slow gait speed and greater fall risk may be largely a byproduct of the fact that age-related decreases in gait speed and increases in fall risk occur in parallel.

Gait variability and stability change similarly for young and older adults with increases or decreases in speed despite older adults having greater average variability and instability (Kang and Dingwell, 2008a,b). Faster walking speed itself has not been associated with decreased stability in young adults and some measures of stability may actually increase with faster walking speed in young adults (England and Granata, 2007; Hak et al., 2013). When only older adults are examined, contrasting results show greater variability in those who walk slower (Verghese et al., 2009) as well as in all older adults regardless of speed (Hausdorff et al., 2001; Dingwell et al., 2017; van Kooten et al., 2018). In a study of individuals with and without diabetic neuropathy, slower speed was a predictor of greater stability in adults with diabetic neuropathy (Dingwell et al., 2000), suggesting that slower walking speed may be used to improve stability in the presence of additional physiological deficits.

The presence of increased gait variability or instability both with and without slowed gait speed in older adults may suggest that, as with decreased muscle strength, slow gait is only used to improve stability once a critical threshold of overall physiological decline is reached. Since walking speed is correlated with muscle strength and cognitive function in older adults (Lauretani et al., 2003; Holtzer et al., 2006), it is unclear whether reduced walking speed itself has a negative effect on balance control, or whether there is a common cause underlying both phenomena. Examining the interacting effects of cognitive function, strength, and walking speed on balance may provide a more complete picture of falls risk.

### 3.4. Increased Visual Dependency

Older adults tend to depend more on visual information compared to younger adults (Osoba et al., 2019). Lord and Webster (1990) first showed that older adults that had experienced a fall recently performed significantly worse on the rod and frame test, indicating increased visual dependence. Jeka et al. (2010) support this result, finding that older adults show increased responses to visual perturbations in standing, with even higher responses in a fall-prone older adult group. Yeh et al. (2014) studied the effect of a secondary cognitive task and time delay in the visual feedback, and found that older adults tend to prioritize visual feedback over proprioception even with a disruptive time delay. More recently, Lee (2017b) studied the relationship between visual dependency, categorized by the rod and disc test, with clinical assessments of balance and vertigo, finding significant differences between visually dependent older adults compared to both young adults and visually independent older adults. A similar study, however, using different clinical tests, found no significant effect of visual dependency, but noted that these tests largely lacked visual components (Lee, 2017a). In contrast, Almajid et al. (2020) do find that performance in the timed up and go test of visually dependent older adults is more affected by a visual perturbation than the performance of visually independent older adults.

In walking, research on the use of visual information has mostly focused on high-level effects like navigation (Warren et al., 2001) and speed control (Lamontagne et al., 2007). Optical flow is also used for balance control during walking (Reimann et al., 2018b), and older adults tend to depend on it more than young adults. Anderson et al. (1998) removed optic flow during walking by occluding a short stretch of the walkway, which led to a significant increase in gait velocity and step length in older adults, but not in young adults. Perturbing optical flow with filtered white noise has a destabilizing effect on both young and older adults, but the effect is generally much stronger in older adults, where it can significantly disrupt step placement (Franz et al., 2015). This difference in the effect of visual perturbations between age groups is substantially more prominent than the effect of a secondary cognitive task or walking with narrow step width (Francis et al., 2015). Qiao et al. (2018a) investigated the effect of perturbed optical flow at joint level and found a general increase in variance that was larger in older compared to young adults. Surprisingly, Qiao et al. (2018b) found a negative relationship between local dynamic instability measures and responses to optical flow perturbations in young adults, and failed to establish any significant relationship in older adults. Kazanski et al. (2020) used optical flow perturbations in a similar paradigm, but failed to find significantly increased visual dependency in the older adult group.

The evidence is relatively clear that visual older adults are more affected by visual perturbations, but there is no clear picture *why* that is the case or *how* these phenomena are linked. One possible explanation is that age-related decreases in muscle strength compromise balance in older adults, and the CNS adapts the gains of the sensorimotor control loop to re-establish robustness. This might explain why Qiao et al. (2018b) found that young people who responded more strongly to visual perturbations tended to have higher local dynamic stability measures. But this pattern did not show up in older adults in the same study, possibly because of the confounding influence of other age-related factors affecting postural stability.

### 3.5. Interactions Between Age Effects and Sensorimotor Control of Balance

There is solid evidence, reviewed above, that cognitive function, muscle strength, and walking speed are correlated with increased fall risk, and that older adults generally depend more on visual information for balance control compared to young adults. But we know little about the causality behind this relationship, of *how* these factors are related, or *why* older adults rely more on vision. Here we briefly review some findings about how walking speed, specifically cadence, and cognition affect the sensorimotor control of balance.

**Figure 2:**
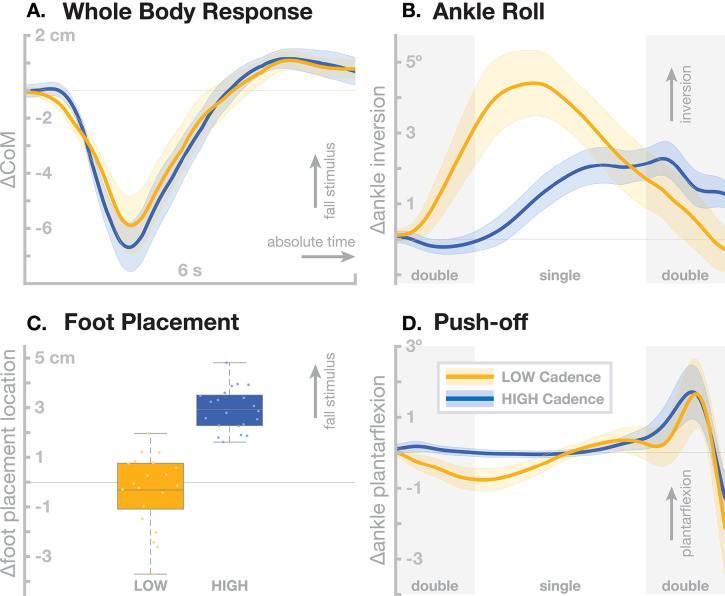
Differences in sensorimotor control of balance depending on stepping cadence. Orange represents walking at *low* cadence (80 bpm), blue at *high* cadence (110 bpm). **(A)** Changes in whole-body CoM over six seconds after the stimulus in absolute time. The other panels show responses in the three balance mechanisms. **(B)** Responses in the ankle roll mechanism over the first step and second double stance after the stimulus in normalized time, with shaded areas marking double stance. **(C)** Differences in foot placement at the first post-stimulus step. **(D)** Responses in the push-off mechanism over the first step and second double stance after the stimulus in normalized time, with shaded areas marking double stance. Color-shaded areas give 95% confidence intervals across subject means, box plots for foot placement cover the inter-quartile ranges, with means marked by horizontal lines, whiskers showing the upper and lower adjacents and dots the individual subject means.

Older adults tend to walk with increased cadence compared to young adults (Judge et al., 1996), even at a reduced average gait speed (Ko et al., 2010). We have investigated the interaction between stepping cadence and sensorimotor control of balance by assessing the effect of artificial fall stimuli in healthy young adults walking while matching their cadence to a metronome at 80 (*low* cadence) or 110 (*high* cadence) beats per minute. Here we summarize the relevant findings from this study. For details, please refer to Fettrow et al. (2019). Figure 2 shows the resulting responses in the whole-body sway and the three balance mechanisms ankle roll, foot placement and push-off. The overall effect of the perturbations is very similar between the two cadence conditions, with no significant difference in the whole-body CoM excursion (Figure 2A). There are substantial differences, however, in the underlying biomechanics of how this whole-body sway is generated by the balance control system. In the *low* cadence condition, participants relied on the ankle roll mechanism significantly more than in the *high* cadence condition (Figure 2B). This relationship was the opposite for the foot placement mechanism, which participants used heavily in the *high* cadence condition, but did not use at a statistically significant level in the *low* cadence condition (Figure 2C). The push-off mechanism, in contrast, was used to a similar degree in both cadence conditions (Figure 2D).

These results show that humans can flexibly choose which balance mechanisms they recruit, depending on a constraint on their gait pattern. In the *high* cadence condition, steps are so frequent that foot placement modulation can be used as the dominant balance mechanism, which might be more metabolically efficient, since muscle force is only used to move the swing foot and the energy for adjusting the motion of the body mass comes from the gravitational field (Kuo, 1999). In the *low* cadence condition, the duration of each stance phase is so long that waiting for the next step to adjust foot placement is less feasible, so the ankle roll mechanism is recruited during single stance. This requires more muscle force than the foot placement mechanism (Kuo, 1999). Furthermore, the force must be generated at the distal ankle joint, rather than the proximal hip joint for foot placement, which older adults tend to favor (Tang and Woollacott, 1999). This cadence effect indicates that the tendency in older adults to walk at higher cadence might be related to their generally reduced muscle strength. By this hypothesis, older adults choose to walk at increased cadence in order to favor the foot placement mechanism for balance control over the less efficient ankle roll mechanism.

Age-related cognitive decline also affects fall risk (Lamoth et al., 2011). Walking requires attention to navigate, steer around obstacles and other people in the environment. Balance control is not considered a primarily cognitive task, the vestibular pathway for balance control might even bypass the cortex completely (Stiles and Smith, 2015). On the other hand, there is a consistent effect of secondary cognitive tasks on balance performance (Horak, 2006). Since cognition is complex, so is, necessarily, the interaction between cognition and sensorimotor control of balance. To begin investigating how cognition affects the balance control system in the control of walking, we combined our balance assessment with a virtual constraint, using a head-mounted display. We added a path to the virtual environment that contained *No-Step* zones, marked in red, and asked participants to not step on the red area. The *No-Step* zones alternated between the left and right of the path, with *Neutral* zones in gray on the other side of the path, so that any fall stimulus would induce a sensation of either falling toward a *No-Step* zone, or a *Neutral* zone. Methods and results from this study are published in detail in Fettrow et al. (2020), and here we briefly summarize the relevant findings.

**Figure 3:**
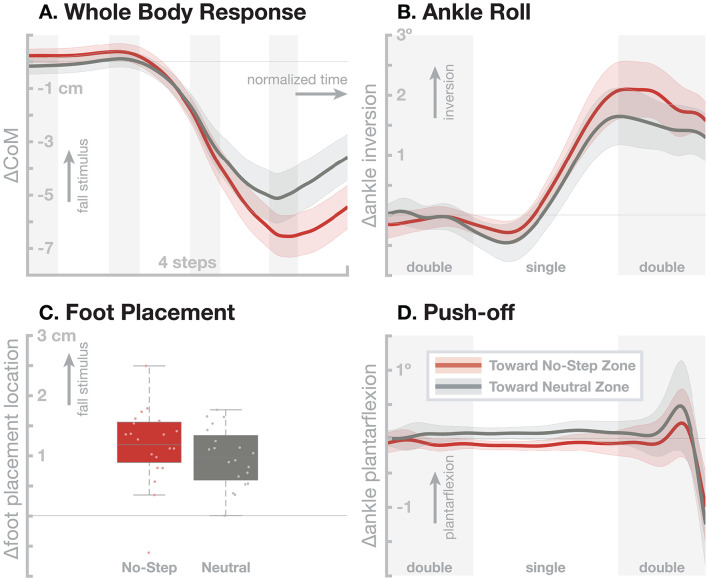
Effect of an added virtual no-step zone on one side of the walking path on the sensorimotor control of balance. Red represents fall stimuli toward a *No-Step* zone, gray represents fall stimuli toward a *Neutral* zone. **(A)** Changes in whole-body CoM over four steps after the stimulus. The other panels show responses in the three balance mechanisms. **(B)** Responses in the ankle roll mechanism over the first step and second double stance after the stimulus, with shaded areas marking double stance. **(C)** Differences in foot placement at the first post-stimulus step. **(D)** Responses in the push-off mechanism over the first step and second double stance after the stimulus, with shaded areas marking double stance. Color-shaded areas give 95% confidence intervals across subject means, box plots for foot placement cover the inter-quartile ranges, with means marked by horizontal lines, whiskers showing the upper and lower adjacents and dots the individual subject means.

Figure 3 shows the resulting responses in the whole-body sway and the three balance mechanisms ankle roll, foot placement, and push-off. The no-step zone has a statistically significant effect on the whole-body sway (Figure 3A), with fall stimuli toward the *No-Step* zone leading to a slightly larger CoM shift than fall stimuli toward the *Neutral* zone. This difference in the whole-body sway is largely generated by a small, statistically significant increase in the foot placement response (Figure 3C). There is was no significant difference in the ankle roll and push-off mechanisms (Figures 3B,D). While the ankle roll is slightly larger for fall stimuli toward the *No-Step* zone, this difference was not statistically significant.

These results show that there is a clear effect of a cognitive task on the sub-conscious balance responses. The overall balance response is larger when the artificial fall stimulus corresponds to a detected deviation of the body toward the no-step zone, bringing it in conflict with the cognitive task. This difference might be due to a dynamic re-weighting of the balance response gains depending on the current location of the no-step zone, or due to an active cognitive component that is superposed over the normal response.

## 4. Understanding the Effects of Different Age-Related Factors on Balance Control

Cognitive ability and muscle strength decline with age, and older people tend to walk slower and rely more on visual information. Each individual factor correlates with age and balance problems, but the factors also correlate with each other. The causal relationship between age, muscle strength, preferred walking speed, visual dependency and balance control is not well-understood. Experimentally modifying individual factors to identify their role in this causal relationship is not straightforward.

One option to understand the effect of each individual factor on the overall behavior is to develop a computational model of the whole system. In such a model, we can then modify individual factors in isolation by changing the specific parameters that describe them, and conduct simulation experiments to observe the result of these modifications on the overall behavior and stability of the system. Developing such a model is, of course, also not straightforward, though feasible in a way that some experimental manipulations are not.

In this section, we introduce an existing neuromechanical model of human walking spanning biomechanics, muscle physiology, spinal reflexes and vestibular control. We then show how this model can be “aged” to study the effect of age-related balance factors, and report the results of a simulation study to isolate the results of common age-related changes in muscle physiology, including reductions in muscle strength.

### 4.1. A Neuromechanical Model of Human Walking

**Figure 4:**
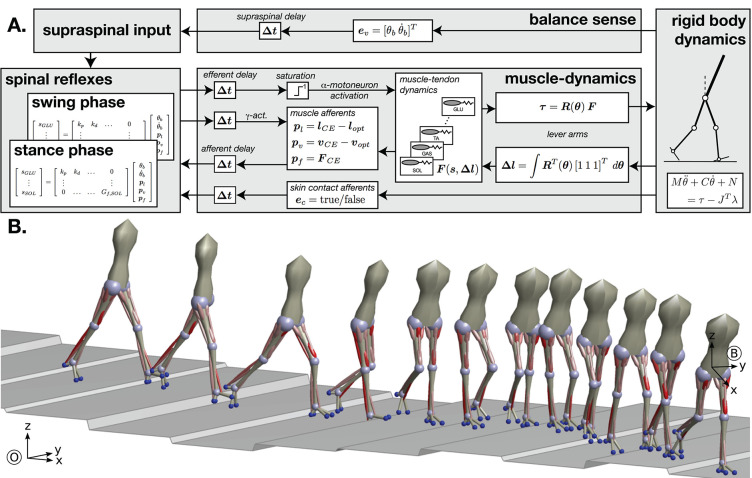
Neuromechanical model of human walking. **(A)** Overview of the model architecture. **(B)** Example of walking behavior generated after neural controller optimization. Red cylinders indicate the leg muscles used in the model, with color saturation signaling the level of muscle activation generated by the controller.

Our neuromechanical model of walking was first introduced for the sagittal plane by Geyer and Herr (2010) and extended to 3d by Song and Geyer (2015). The model activates muscles based on a list of 10 explicit reflex modules that directly link various sensory inputs to muscle activation. Walking movement patterns emerge from the interaction of the muscle forces from these reflex modules and the reaction forces from ground contact. Figure 4 shows an overview of the model architecture and a sample walking pattern generated by the model. The model has previously been used to predict human perturbation responses against different perturbations (Song and Geyer, 2017) and to model age-related changes in human walking performance (Song and Geyer, 2018).

The mechanics consists of seven rigid body segments (trunk, thighs, shanks, and feet) and eight degrees of freedom (hip pitch and roll, knee pitch, ankle pitch). The rigid body mechanics interact with the muscle dynamics through geometric conversion of joint angles (θ) into muscle lengths (Δ*l*) and of muscle forces (F) into joint torques (τ). The instantaneous moment arms of the muscles are captured in the matrix R(θ). The matrix is diagonal except for a few off-diagonal terms accounting for the biarticular nature of some leg muscles. The length changes Δ*l* together with the muscle stimulations *s* form the input for the computation of the muscle tendon dynamics *F*(*s*, Δ*l*), which are modeled as Hill-type muscles. Hill-type muscles models combine an active, force-generating element with passive parallel and serial elastic elements, generating dynamics similar to muscle-tendon units. We model major leg muscles that are involved in human gait, including the monoarticular soleus, tibialis anterior, biceps femoris short head, vastus group, gluteals, and combined hip flexors, as well as the biarticular gastrocnemius, hamstrings, rectus femoris, and the hip abductors and adductors, for a total of 11 functional muscles at each leg. Besides the forces *F*, the contractile elements (CE) of the muscle tendon dynamics also generate proprioceptive signals from the muscle spindles (*p*_*l*_ and *p*_*v*_) and the Golgi tendon organs (*p*_*f*_) carrying information about the muscle length, velocity, and force. Although more complex models of these sensory organs exist, they are reduced to proportional signals with offsets for the length and velocity in this particular model.

The reflex control layer receives a range of sensory inputs and generates the muscle stimulations (α-motorneurons) and fusimotor drives (γ-motorneurons). The sensory inputs include the proprioceptive signals from the muscle dynamics and exteroceptive signals from the rigid body mechanics (*e*_*v*_ and *e*_*c*_). The latter represent the high-level balance sense based on the visual and vestibular systems providing information about the upper body orientation (θ_*b*_) and the mechanoreceptors providing information about the environment interaction in the form of contact detection and ground reaction forces. Note that in neuromechanical gait models the sensory organs for exteroception are generally modeled with less detail than proprioceptors. The estimate of the upper body estimation determines the target angle of the swing leg at heel-strike, generating larger steps when the body leans more. This feedback principle is similar in effect to the balance control scheme proposed by Hof (2008), but with more a complex body geometry, segment angles are more robust state variables than foot location and is more commonly used in robotics (Yin et al., 2007; Wang et al., 2012). The sensory pathways as well as the motor pathways interfacing the spinal α-motorneuron pools and the mechanical layers are time delayed (Δ*t*), mimicking the signal transmission delays in the sensory and motor axons.

The synaptic interconnections between sensory inputs and motor outputs that form the reflex control of the different muscles in the spinal α-motorneuron pools are based on ten functional control modules which embed key functions of legged systems. The modules are organized functional groups that control the stance and swing legs. Key functions of the stance leg control modules are the generation of compliant, spring-like leg behavior, the prevention of knee hyperextension, balancing of the trunk, compensation of the swing leg interactions and flexion of the ankle to prevent ankle overextension. Swing leg modules provide ground clearance of the swing foot and move the leg to a specific target configuration. Individual swing leg modules generate ankle flexion, hip swing, and knee stabilization during the early swing phase, and decelerate and stabilize the leg in the late swing phase. In Figure 4, the synaptic interconnections in these reflex modules are represented by matrix multiplications. While this linear representation is accurate for many of the modeled reflexes, the model has more complex interconnections as well. For instance, some reflexes use multiplication of several inputs similar to presynaptic inhibition. Other reflexes include nonlinear effects, such as the switching between stance and swing reflex connections due to input from the mechanoreceptors.

### 4.2. Modeling the Effects of Aging on Sensorimotor Control of Balance

Here we demonstrate how such a mechanistic, predictive model can be used to test hypotheses about the effect of specific factors on balance control. Specifically, we use the model to investigate the effect of a list of known age-related physiological changes, most prominently loss of muscle strength, on sensorimotor control of balance. As seen in section 3.2, loss of muscle strength is associated with increased fall risk, but we do not know if loss of muscle strength directly causes increased fall risk, or if the two phenomena are only correlated. Here we demonstrate how a neuromechanical model can be used to investigate the causal relationship between muscle strength and fall risk. For simplicity, we analyze the much narrower hypothesis that decreased muscle strength causes changes in the sensorimotor feedback control of balance. This hypothesis predicts that the model with weaker muscles would show systematic differences in the feedback law mapping sensory input to motor output for balance. To test this prediction, we ran a model simulation study using two populations of young and old models.

#### 4.2.1. Age-Related Physiological Changes

We modify the parameters representing properties of the skeleton, muscles and the nervous system to represent an ≈80 year old human, compared to the basis model that represents a ≈20 year old human. This “aging” process is done by adapting a sub-set of the physiological parameters in the model, following Song and Geyer (2018) and explained in more detail below. The model used here is a combination of different versions of the model published previously by Song and Geyer (2015, 2018). Both of these models build upon the earlier work by Geyer and Herr (2010), which showed that simple reflex modules can generate walking movement in the sagittal plane. The Song and Geyer (2015) work extended this to 3d, adding lateral degrees of freedom at the hip and an associated list of reflex modules. The Song and Geyer (2018) work extended the model by “aging” the physiological parameters and adding noise, mainly to investigate the relationship between age and walking speed, metabolic cost and fatigue. It did not contain balance control in 3d, since it was still constrained to the sagittal plane. The study reported here combines these two directions in a model that includes both balance control in 3d and “aged” physiology to investigate interactions between these.

The most prominent age-related change we model is a reduction of muscle strength by 30% and muscle contraction velocity by 20%. Beyond that, we increased eccentric force enhancement by 30% and excitation-contraction coupling time by 20%. Skeletal modifications comprise changes in the body mass distribution and in the range of motion the hip. To model the loss of leg muscles and gain of body fat, we reduced leg mass by 10% and increased trunk mass accordingly to keep the total body weight unchanged. We reduced hip motion range by 20% due to muscle contracture. Neural modifications include an increase of the transmission delays by 15%. Further implementation details can be found in Song and Geyer (2018).

#### 4.2.2. Optimization

We obtain control parameters using the covariance matrix adaptation evolution strategy (Hansen, 2006). For each individual model, we optimize the behavior with respect to the cost function that consists of three parts:

(1) $$J=\{\begin{array}{ll} 2c_{0}-x_{\text{fall}} & \text{if fall}\\ c_{0}+d_{\text{steady}} & \text{if non-steady walk}\\ 100||v_{\text{avg}}-v_{\text{tgt}}||+d_{\text{steady}}\; & \text{else}, \end{array}$$

with *c*_0_ = 10^3^ and *d*_steady_ as a steadiness measure summing up the differences of relative positions of the body segments at touchdown. The first part of the cost function generates basic walking without falling. The second part generates steady locomotion and the third part adjusts the steady walking to a desired movement speed. A more detailed description of the optimization process can be found in Song and Geyer (2015).

Since the optimization process is stochastic, each optimization results in a different individual model. We repeated the optimization to generate populations of multiple *young* and *old* models, with a size of *N* = 9 individuals in each group, targeting a walking speed of 1.3 m s^−1^. The sample size was limited by the processing time of the evolutionary parameter optimization, which took two to three days for a single individual.

#### 4.2.3. Simulation Study

The optimization process resulted in two populations of *young* and *old* models. The only explicit difference between the models was in the list of age-related changes to physiological parameters specified above. It is possible, though, that the optimization process results in additional implicit differences between the populations that emerge from the evolutionary pressure encoded in the cost function (Equation 1).

We simulate a study using artificial fall stimuli on *young* and *older* participants. We model the fall stimulus in the form of a bias to the sensory signal for the trunk roll angle of 0.05 rad for a duration of 0.6 s. The magnitude of the modeled stimulus was chosen to generate a foot placement response that was approximately in the same range as the average response of human subjects to visual stimuli of 60°s^−1^ (Reimann et al., 2018b) The perturbation is applied at left heel strike. We then compare the behavior of the perturbed with the unperturbed models and measure the responses to the perturbations by subtracting the unperturbed from the perturbed trajectories, analogous to the processing of experimental data (Reimann et al., 2018b).

We analyze the effect of aging on sensorimotor control of balance in the model statistically in a similar way to how we analyze experimental data (Reimann et al., 2018b). We use t-tests on the difference between the whole-body CoM at the end of the four steps and the foot placement change at the first post-stimulus step, with α = 0.05. We additionally performed an F-test of equality of variances for the foot placement response, to test the hypothesis that older adults show increased variability under sensory perturbations.

#### 4.2.4. Results

**Figure 5:**
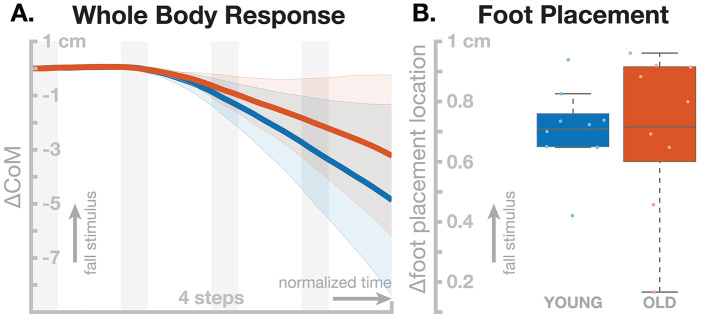
Effect of aging in the neuromechanical model parameters on the sensorimotor control of balance. Blue represents the *young* model, red the *old* model. **(A)** Changes in whole-body CoM over four steps after the stimulus. **(B)** Differences in foot placement at the first post-stimulus step. Color-shaded areas in **(A)** give 95% confidence intervals across the individual parameter sets, box plots in **(B)** cover the inter-quartile ranges, with means marked by horizontal lines, whiskers showing the upper and lower adjacents and dots the individual data points.

All individual models were able to withstand the effect of the sensory perturbation for at least four steps without falling, after which we stopped the simulations. Figure 5 shows the motor responses generated by the *young* and *old* model populations. The shift in whole-body CoM over four steps was slightly larger in the *young* model group (Figure 5A), but this difference was not statistically significant, *t*_(8)_ = 1.4795, *p* = 0.1773. The models showed active responses in the foot placement mechanism, shown in (Figure 5B) Visual inspection shows that both the whole-body and the foot placement responses are similar between the two groups, and the *t*-test support this by not reporting statistical significance for the foot placement [*t*_(8)_ = 0.0630, *p* = 0.9513]. The *old* model population shows increased variance in the foot placement mechanism compared to the *young* models, but this difference also failed to reach statistical significance [*F*_(8, 8)_ = 3.4613, *p* = 0.0983].

## 5. Discussion

Older adults fall more often than young adults, but the causes for this increase are still not well-understood. One knowledge gap is our lack of understanding of the neural processes for the sensorimotor control of balance during walking, that map sensory information to appropriate motor responses to maintain balance. Another knowledge gap is that we do not understand how multiple different age-related factors that are correlated with balance control and fall risk, interact with each other and the neural control process for walking.

Here we reviewed research that attempts to overcome these knowledge gaps. Our own recent work uses artificial fall stimuli to characterize the sensorimotor control system for balance during walking by observing how it maps sensory input to motor output. In a literature review, we gathered knowledge about how the age related factors of cognitive decline, muscle weakness, gait speed and increased dependency on visual information is correlated with balance control and fall risk. We argued that the appropriate method to understand how all of these factors interact is to use computational models that formalize the existing knowledge. These models allow concrete predictions, that can then be tested experimentally. We introduced one such model and used it for a simulation study that isolates the effect of several age-related neurophysiological changes, most prominently muscle weakness.

The simulation study resulted in no statistically significant differences between the *young* and *old* model populations. This result does not support the narrow hypothesis that age-related muscle weakness causes differences in the sensorimotor feedback control of balance in walking. The more general hypothesis that age-related muscle weakness causes increased fall risk is, of course, harder to analyze and would require, among other things, a functional measurement of “fall risk” for the model. While it seems straightforward that the sensorimotor control balance affects fall risk in some way, we do not know the details of this relationship, and more research is needed to understand it.

### 5.1. Limitations in the Experimental Approach

While measuring how the sensorimotor control system for balance responds to fall stimuli is an important first step to understand this system, it is limited to observing the kinematic, kinetic, and electromyographic responses at the surface, and does not attempt to observe the underlying neural dynamics. We essentially treat the nervous system as a black box. This is a practical limitation, as most imaging techniques have constraints that severely limit their application to study walking. Other researchers, however, have started to chip away at these constraints. Motion artifacts in EEG systems can be avoided by both software and hardware approaches (Peterson and Ferris, 2019). While movement of any kind is still a challenge for FMRI recordings, a number of studies have attempted to circumvent this obstacle and image brain activity during balance-related activities (Papegaaij and Hortobágyi, 2017; Wittenberg et al., 2017). These promising efforts should be seen as valuable alternate approaches that are complemented by the biomechanics and motor control techniques described here.

### 5.2. Limitations in the Model Approach

The current neuromechanical model is, of course, limited. Biomechanically, it lacks several important degrees of freedom, most notably the subtalar joint for ankle inversion and eversion, which is required for the ankle roll mechanism. Also lacking is internal rotation at the hip, a bendable spine, and arms. While the role of these degrees of freedom is more subtle, they are undeniably used in balance control.

On the neural control level, the rhythmic activation patterns in the model are generated by dedicated reflex modules, with a large number of 82 parameters that are determined using evolutionary optimization. Reflexes are without a doubt important in maintaining stability, particularly force feedback has been shown to be important for generating spring-like compliant behavior during the stance phase in many animals (Duysens et al., 2000). Other reflex modules are less directly inspired by physiological observations and were introduced based on functional biomechanical principles to generate walking behavior (Geyer et al., 2006). While the resulting model does successfully walk, it largely lacks the flexibility to adjust the walking movement patterns in goal-directed ways. The current model cannot freely modulate its walking speed, change direction, or modulate step width or length. While it is possible to add other structures that solve some of these issues, like central pattern generators (Dzeladini et al., 2014; Van der Noot et al., 2018), the hard-wired, dedicated reflex modules solving one particular task are fundamentally at odds with the seemingly effortless flexibility of human movement (Duysens and Forner-Cordero, 2018). While the current model is an impressive demonstration of how far purely reflexive movement generation can go, successfully modeling the full range of human motor control will likely require a different, more flexible approach.

The limitations in the model presented here are in contrast with models used in studies to investigate the effect of weakness, contracture and activation limits of specific muscles associated with neuromotor impairments such as stroke or cerebral palsy (e.g., Steele et al., 2012; Knarr et al., 2013; Pitto et al., 2017; Fox et al., 2018; Kainz et al., 2018). These models are generally physiologically more detailed, with more biomechanical degrees of freedom and muscles actuating them. The model is then used to estimate the muscle activation that generated experimental behavior, recorded by a combination of motion capture, force plate, and electromyographical data, using inverse kinematics, inverse dynamics and optimization (Thelen et al., 2003). This modeling approach does not deal with balance, since the balance problem was already solved by the human during the experimental session where the data was recorded. More generally, this model type does not ask questions of sensorimotor feedback control, since the approach of fitting previously recorded experimental data means that the control is by definition open-loop and cannot use sensory data to modify the control signal. This direction of modeling is, in that sense, orthogonal to the work we presented here, with both approaches are designed to answer specific questions. Ideally these two directions can be combined at some point to models that are biomechanically and physiologically detailed and capable of both generating behavior using closed sensorimotor feedback control loops and explaining observed experimental data by fitting underlying control laws and motor plans to data.

### 5.3. The Role of a Computational Model for Generating Knowledge

What have we learned from the simulation study performed here, and what can we learn from computational models in principle? The simulation failed to show any differences between the *young* and *old* model populations. Arguing that this is a failed experiment, however, would miss the larger point of understanding a complex system with many moving parts. We analyzed the narrow hypothesis that age-related decline in muscle strength causes changes in the sensorimotor feedback control of balance. Extending this result to fall risk would require a link between fall risk and sensorimotor control of balance. One possibility for such a link is that muscle weakness drives adaptive changes in feedback control, where weaker muscles decrease stability and the feedback control system adapts by increasing gains to re-establish robust balance. The adaptation process is implicitly modeled by the parameter optimization process, which has a measure of stability as part of the cost function (see Equation 1). The simulation study did not result in increased sensorimotor feedback gains in the *older* model, refuting the hypothesis within the limits of the assumptions. One such limit is that the stability requirement in the cost function is relatively mild, consisting only of a term that rewards body similarity in body configurations between gait cycles. A more robust accounting for stability could add various perturbations and reward robust responses. This hypothesis would also imply that sensorimotor feedback gains in older adults are actually increased. While there is a body of corroborating evidence, this prediction should still be directly tested.

This hypothesis makes intuitive sense, since reduced muscle strength limits the range of perturbations one can successfully recover from. However, rehabilitation programs for balance control targeting muscle strength have had mixed success, with best results for programs that include muscle strength training in a multi-factor approach (Horlings et al., 2008). These results imply that other factors also play a role. The simulation study here had, essentially, the same result, that muscle weakness *alone* fails to explain the observed differences in balance control between young and older adults.

A model is a form of formalized reasoning. While this model is limited, it is important to point out that other ways of generating conclusions are also limited (Smaldino, 2017). The argument chain that (A) older people have weaker muscles, and (B) weaker muscles increase fall risk, so we understand why (C) older people have increased fall risk is clearly not air-tight. This argument chain is also a model, in the sense that it draws conclusions about underlying causes from observable facts. However, such a verbal model contains vague definitions and implications. Formalized, computational models also have underlying assumptions, but they have the benefit that it is possible to bring these assumptions out into the open and discuss them explicitly. Often the attempt to formalize a complex system in a model forces us to go through this process of making our hidden assumptions explicit, allows us to study their validity and effect on the system and ultimately improves our understanding of the system as a whole.

## Data Availability Statement

The datasets generated for this study are available on request to the corresponding author.

## Author Contributions

HR, RR, JH, TF, and JJ wrote the manuscript. RR, HR, and HG developed the model. RR and HR designed and performed the simulation studies.

## Conflict of Interest

The authors declare that the research was conducted in the absence of any commercial or financial relationships that could be construed as a potential conflict of interest.
